# Clinical Outcomes and Their Prognostic Factors among Cervical Cancer Patients with Bone Recurrence

**DOI:** 10.1155/2022/3446293

**Published:** 2022-09-10

**Authors:** Thiti Atjimakul, Jitti Hanprasertpong

**Affiliations:** ^1^Department of Obstetrics and Gynecology, Faculty of Medicine, Prince of Songkla University, Songkhla 90110, Thailand; ^2^Department of Research and Medical Innovation, Faculty of Medicine Vajira Hospital, Navamindradhiraj University, Bangkok 10300, Thailand

## Abstract

**Background:**

Bone recurrence occurs in 0.75%-8% of cervical cancer patients after primary treatment. Only a few previous studies have reported on survival times associated with prognostic factors for bone recurrent cervical cancer. This study aimed to evaluate the oncological outcomes and their predictors among cervical cancer patients with bone recurrence.

**Methods:**

The medical records of cervical cancer patients with bone recurrence who received primary treatment at Songklanagarind Hospital from January 2002 to December 2017 were retrospectively reviewed. Prognostic factors were identified using a Cox regression model.

**Results:**

The study included 6,354 cervical cancer patients, of whom 98 (1.54%) had bone recurrence at a median time of 25 months after the primary treatment (range 4.9-136 months). The most frequent site of bone recurrence was the spine (81.00%); the two most common visceral coexisting recurrence sites were the lungs and the liver. The median recurrence-free interval (RFI) was 21 months. Of the patients with recurrence, 75 (76.50%) were treated with combined radiation therapy and chemotherapy. The one-year overall survival (OS) after recurrence was 22.70%. On multivariate analysis, age under 60 years at the time of recurrence diagnosis (hazard ratio [HR] = 2.48, 95% CI = 1.47-4.18, *p*=0.001) and an RFI less than 21 months (HR = 1.63, 95% CI = 1.04-2.55, *p*=0.03) were independent prognostic factors for OS after recurrence.

**Conclusion:**

Bone recurrence in cervical cancer patients is rare and is associated with poor survival. Our study found that age and RFI were significant prognostic factors for OS in cervical cancer patients with bone recurrence.

## 1. Introduction

Cervical cancer is a significant global health problem, as it is the fourth most frequent cancer among women. It remains the leading cause of cancer death in women, particularly in less-resourced countries [1]. Treatment of cervical cancer depends mainly on the stage of the disease. Management may include surgical treatments such as simple hysterectomy or radical hysterectomy, radiotherapy, and/or chemotherapy [2–4]. Over the past couple of decades, although advances in imaging technology, surgical techniques, radiotherapy, and chemotherapy have led to improvements in both disease control and survival, substantial treatment failures still occur in this patient group. The recurrence rate by stage is as follows: for the International Federation of Gynecology and Obstetrics (FIGO) stage IB around 10%, for stage IIA 17%, for stage IIB 23%, for stage III 42%, and for stage IVA 74% [4, 5]. Most cervical cancer recurrences occur within 2-3 years after primary treatment [2–4].

A distant (metastatic) recurrence means the cancer has traveled to a distant part of the body and indicates that cancer cells have already spread through the hematogenous or lymphatic pathways. The most common sites of distant recurrence are the nonregional lymph nodes, lungs, liver, and bone [6, 7]. Bone recurrence occurs in 0.75%-8% of cervical cancer patients after primary treatment [7–13]. The current treatment for cervical cancer patients with bone recurrence is complicated and may involve surgery, chemotherapy, and/or bone radiation depending on the primary treatment, recurrence site, recurrence-free interval (RFI), recurrence symptoms, and performance status [5, 14]. Patients with a bone recurrence typically have a very poor prognosis, and the 5-year overall survival (OS) after bone recurrence is only around 10% [7, 9].

Several previous studies have identified prognostic factors after recurrent cervical cancer as shown in the pattern of recurrence, symptom status, white blood cell (WBC) count [2], SCC Ag after recurrence, high metastatic burden [6], histologic type, pelvic node status at initial surgery, mode of salvage treatment [9], size of recurrence, secondary radical surgery [12], FIGO stage, and the number of recurrence sites [15].

Only a few previous studies have reported on survival times associated with prognostic factors for bone recurrent cervical cancer [10, 11, 16, 17]. A better understanding of survival and the factors involved in cervical cancer with bone recurrence would help physicians inform and advise patients appropriately. Therefore, we performed a retrospective review to investigate the clinical characteristics and treatment results for patients with cervical cancer and bone recurrence and to identify associations with clinical outcomes.

## 2. Materials and Methods

The institutional review board of the Faculty of Medicine, Prince of Songkla University approved this study. Of 6,354 patients with cervical cancer registered in our hospital from 2002 to 2017, 98 (1.5%) patients received primary treatment at our hospital and later developed bone recurrence. All bone recurrence diagnoses included in the study were confirmed by histology or imaging diagnosis. Patients who were known to have other cancers or had incomplete medical information were excluded. The patients in the study had cervical cancer staging based on the 2009 FIGO criteria as IA to IVA. All patients received the standard primary treatment in our institution depending on the FIGO stage. Since 2000 at our hospital, cervical cancer has been treated with CCRT with cisplatin for locally advanced (FIGO stage IIB to IVA) disease. However, there were still a group of patients with locally advanced disease who did not receive it due to various reasons such as personal refusal, old age, poor performance status, having other comorbid diseases, or a poor economic situation. Surgery treatment was considered for women diagnosed in the early stages (FIGO stage IA-IIA) of the disease with or without postoperative adjuvant treatment, and conventional radiation or CCRT was offered as an alternative in cases where a patient was not fit for surgery or the patient requested nonsurgical treatment. Neoadjuvant chemotherapy (NACT) followed by radical surgery may have been used as an alternative treatment in bulky stage IB2-IIA cervical cancer cases. Our post-treatment surveillance protocol consisted of a follow-up visit every 3 months in the first year, every 4 months in the second year, every 6 months in the 3rd to 5th years, and annually thereafter [2].

During the study period in our institution, bone recurrence following cervical cancer incidentally discovered during a routine follow-up visit in a patient without relevant complaints was considered asymptomatic. A recurrence was classified as symptomatic if the patient complained of relevant symptoms prior to a physical and/or gynecologic examination. A suspected bone recurrence was confirmed by positive findings from an imaging modality such as X-ray, bone scan, computed tomography (CT), or magnetic resonance imaging (MRI) or pathologically by bone biopsy.  Table 1 summarizes the clinical characteristics of the study patients diagnosed with bone recurrence of cervical cancer such as age at the time of recurrence, initial stage, histological cell type, the primary treatment for the cervical cancer before the recurrent disease, and the clinical characteristics at the time of recurrence, including symptoms, investigations for diagnosis of recurrence, pattern of tumor spread, recurrence sites, coexisting recurrence, and treatment for the recurrence. WBC count, hemoglobin (Hg) level, and platelet (Plt) count were obtained from the patients' records at the initial diagnosis of bone recurrence. The main symptoms leading to the diagnosis of bone recurrence were bone pain, paralysis, or bone fracture. On radiological imaging, the type of tumor spread was divided into two categories: solitary metastasis or multiple metastases; multiple metastases were diagnosed when more than two lesion sites were found. The anatomical distribution of the bone recurrence sites was divided into three categories, limited to the pelvic bone, nonpelvic bone, and a combination of pelvic bone and nonpelvic bone. Coexisting recurrences were categorized by visceral organ involvement, such as lung, liver, brain, and/or nonregional lymph node (such as para-aortic, inguinal, supraclavicular, and/or mediastinal) metastasis. Treatment for bone recurrence was determined by a gynecologic oncologist, radiation oncologist, and/or the surgeon following current practices alongside hospital treatment guidelines and considering the patient's previous treatment, site of recurrence, coexisting recurrence (s), and performance status. The patients were grouped according to their recurrence treatment, divided into radiotherapy, chemotherapy, combined treatment, or supportive care groups. RFI was defined as the time from the end of the initial treatment to the diagnosis of recurrence. OS after recurrence was defined as the time from the recurrence diagnosis to death from cancer-related death or alive with disease at the final follow-up. Clinical characteristics data are presented as frequencies and percentages. Recurrence survival was plotted using the Kaplan–Meier method and the log-rank test for statistical significance. Multivariate analysis using Cox proportional hazards regression was used to determine the independent prognostic factors for OS after recurrence. Results with a *p*-value < 0.05 were considered to be statistically significant. Data were analyzed using STATA version 17 (StataCorp, College Station, TX, USA).

## 3. Results

During the study period, 98 cervical cancer patients had bone recurrence with a median time of 25 months after their primary treatment (range 4.9-136 months). The mean age at diagnosis of bone recurrence was 53 (±12) (range 32-79) years. The patient characteristics shown in Table 1 indicate that bone recurrence was mainly found in stage III at initial diagnosis (42.9%). Most patients had SCC diagnosis (82.6%), and 48% had received CCRT as their primary treatment. Almost all recurrence patients were symptomatic (95%), and more than 85% of the recurrence patients presented with bone pain. Most patients had skeletal scintigraphy (70.4%) alone or combined with other radiologic tests to diagnose the recurrence. Only 8.2% required confirmation by biopsy. At presentation, 62.2% had a solitary bone lesion while 37.8% had multiple bone lesions. Most bone recurrences were nonpelvic bone lesions (73.5%), with the most common site the lumbar spine (53.1%), followed by the pelvis (26%) and thoracic spine (21.4%). At the time of recurrence, 54 patients (55.1%) had a coexisting organ recurrence. The lungs and liver were the two most common covisceral metastatic sites. The most common treatment for the bone recurrence was radiation therapy (55.1%) (10 fractions at 300 cGy/d) at the recurrence sites. Twenty patients opted for only the best supportive care available at our institution.

The median follow-up time after the recurrence was 25 months. 72 patients (73%) died from cancer within one year of the recurrence being diagnosed. The median recurrence survival was six months. The 1-year OS after the recurrence was 22.7% (95% CI = 15%-32%). Univariate analysis of the clinicopathologic prognostic factors and median OS after bone recurrence (Table 2) found that age, FIGO staging, symptomatic recurrence, leukocytosis, and thrombocytosis were usefully associated with poorer OS after bone recurrence. Multivariate analysis revealed that age less than 60 years at the time of bone recurrence diagnosis (HR = 2.48, 95% CI = 1.47-4.18, *p*=0.01) and RFI ≤21 months (HR = 1.63, 95% CI = 1.04-2.55, *p*=0.03) were independent adverse prognostic factors for median OS after bone recurrence (Table 3).

## 4. Discussion

The incidence of bone recurrence rates differs widely depending on the method of detection [10, 14, 16]. Diagnostic imaging techniques such as bone scan, CT, MRI, and 18FDG-positron emission tomography are widely accepted for use in the diagnosis of bone metastasis. However, false positives from all of these imaging techniques have been reported in several previous studies [10, 14, 18]. In addition, previous studies have shown that the rates of bone metastasis in living patients are lower than at autopsy [10, 16, 19]. In our study, only 8.2% of the patients required bone biopsy for confirmation of metastasis. So, these results do not rule out the possibility of other types of bone lesions in the nonbiopsied patients. We had, and still have, the policy of not obtaining a bone biopsy of every patient with suspected bone metastasis. Bone biopsy is an invasive procedure, and histological confirmation is considered necessary only if it is critical for decisions regarding further management when the imaging results are equivocal or not consistent [18].

Our study also found that bone recurrences usually presented symptomatically, with 95% of the bone recurrence cervical cancer patients symptomatic with bone pain or neurological deficit, which is consistent with previous studies which found 62-93% of bone recurrence patients had bone-related symptoms [10, 11, 16]. Therefore, the physician should look for evidence of bone recurrence if a patient has any suggestive symptoms. However, bone investigations after primary treatment in asymptomatic patients with cervical cancer are not normally indicated for routine follow-up, and to date the evidence base justifying such investigations is limited, as also recommended in a Cochrane review of follow-up protocols for cervical cancer patients after primary treatment [20].

We found that the most common site of nonpelvic bone involvement was the axial skeleton area, such as the lumbar spine, the most frequently involved area (53.1%), which is the same as in a previous report [10]. Nonpelvic bone recurrence near the area of a primary tumor is reflective of the efficacy of the primary treatment, whether radiation therapy or concurrent chemoradiation therapy covering the pelvic area only [10, 17]. Yoon et al. [17] studied the clinical characteristics of patients who had bone recurrence following primary treatment of cervical cancer and found the most coexisting metastatic site recurrence was the lungs, which was similar to our study which found visceral organ (lung and liver) involvement in 55.1% of our cervical cancer with bone recurrence patients. This implies that hematogenous spreading could be the primary route of bone metastasis [21].

The treatment of bone recurrence cervical cancer remains a challenging clinical problem. Despite recent advances in aggressive management through multimodal therapy, the median survival after the diagnosis of bone recurrence cervical cancer was only 4-12 months in recent previous studies [8, 10, 11, 17, 22], which the 6 months in our study was consistent with. In previous studies, certain clinicopathologic variables have been found to be related to a poor prognosis for bone recurrence cervical cancer patients, that is, the presence of nonpelvic bone metastasis [10], extra-osseous metastasis [16], adenocarcinoma [17], advanced stage (IIB-IV) [17], multiple initial bone metastases [17], and age less than 45 years at the time of the initial cervical cancer diagnosis [11]. We found only age under 60 years at the time of bone recurrence diagnosis and an RFI less than or equal to 21 months were independent prognostic factors for OS after bone recurrence.

Inflammatory responses such as leukocytosis and thrombocytosis caused by the cancer might play important roles in tumor development, including cancer initiation, promotion, invasion, and metastasis at various stages [2, 23, 24]. Several previous studies including a systematic review and meta-analysis have reported a prognostic role for inflammatory markers on oncological outcomes of patients with cervical cancer [2, 23–25]. Hanprasertpong et al. [2] reported that pretreatment WBC count was found to be a prognostic factor in recurrent cervical cancer after radical hysterectomy. In our study, we found that patients with pretreatment leukocytosis or thrombocytosis had a significantly increased risk of worse OS in univariate analysis. However, after adjusting for other factors, neither were found to be independent adverse prognostic factors. This result may be explained by the fact that the number of patients in our study may not have been sufficient to detect the effect of inflammatory responses on clinical outcomes.

Age less than 60 years at the time of bone recurrence diagnosis was associated with poorer patient survival after bone recurrence in this study, which is similar to the study of Nartthanarung et al. [11], which found that young patients with bone metastasis aged less than 45 years at the time of cervical cancer diagnosis had poorer survival than older patients. Possible explanations for this might be different patient characteristics or different human papilloma virus (HPV) genotype distributions [11, 26]. Serrano et al. [26] ascribed more aggressive behavior of cervical cancer in younger women to a different HPV genotype distribution. This is an important issue for future research.

One interesting finding of our study was that an RFI less than or equal to 21 months was associated with poorer patient survival after bone recurrence. This finding is in agreement with Duyn et al.'s findings that patients with cervical cancer who achieved complete remission after complete primary treatment with an early recurrence had worse prognoses. They found that the relative risk of death was 0.70 per year DFI (95% CI: 0.48-1.00) [27]. The physician should advise patients on the symptoms and warning signs of bone recurrence and also be alert to signs of recurrence in following up recovered cancer patients, especially when a patient visits a surveillance program which may enable early detection of recurrence with appropriate investigations and treatment to improve the outcome of recurrence patients [28, 29].

This study had some limitations. First, as it was a retrospective study, confounding biases might have been missed in the analysis. Another limitation is that the data were from cases at a single institute with limited patient data; therefore, selection and time-trend biases were inevitable. In addition, the treatment of bone recurrence might have been influenced by the previous primary treatment, the patient's health status, and/or the attending physician's preferences. Further studies are required concerning possible surveillance techniques after primary treatment of cervical cancer such as novel imaging or screening for possible markers to identify patients with cervical cancer who have a high risk of bone recurrence.

In summary, our study provides supportive evidence that bone recurrence in cervical cancer patients is rare and is associated with poor survival. Age and RFI were independent prognostic factors for survival in this study.

## Figures and Tables

**Table 1:** Clinical characteristics of study patients diagnosed with bone recurrence cervical cancer.

Characteristic	*n*	(%)
FIGO (2009)		
I	10	10.20
II	39	39.80
III	42	42.90
IV	7	7.10
Histopathologic diagnosis		
Squamous cell carcinoma	81	82.60
Adenocarcinoma	11	11.20
Adenosquamous carcinoma	3	3.10
Other		
Primary treatment		
Radiation	45	45.90
Concurrent chemoradiation	47	48.00
Surgery	6	6.10
Symptomatic		
No	5	5.10
Yes	93	94.90
Investigation		
Plain radiography	12	12.20
Skeletal scintigraphy	69	70.40
Computer tomography	50	51.00
Magnetic resonance imaging	28	28.60
Tissue biopsy	8	8.20
Tumor spread		
Solitary	61	62.24
Multiple	37	37.76
Site of bone recurrence		
Pelvis	12	12.24
Nonpelvis	72	73.47
Both	14	14.29
Coexisting recurrence		
No	42	42.86
Yes	56	57.14
Organs with coexisting recurrence		
Lymph nodes	38	38.90
Lungs	18	18.40
Liver	17	17.30
Brain	1	1.00
Combination	18	18.40
Treatment of bone recurrence		
Radiation	54	54.10
Chemotherapy	7	7.14
Radiation-chemotherapy	14	14.29
Supportive care	20	20.41
Surgery with radiation with chemotherapy	3	3.06
Leukocyte count (*μ*L)		
<10000	29	29.60
≥10000	69	70.40
Hemoglobin (g/dL)		
<11	42	42.90
≥11	56	57.10
Platelet count (*μ*L)		
<400000	32	32.70
≥400000	66	67.30

FIGO stage, International Federation of Gynecology and Obstetrics Staging System 2009.

**Table 2:** Univariable clinical prognostic factors for overall survival.

Factor	*n*	Median (months) (95% CI)	*p*-Value
Age at diagnosis of bone recurrence (years)			
<60	68	5 (3-7)	0.001
≥60	30	11 (5-26)	
FIGO (2009)			
I	10	5 (0-100)	0.031
II	39	5 (3-7)	
III	42	7 (4-9)	
IV	7	35 (1-100)	
Histopathologic diagnosis			
Squamous cell carcinoma	81	7 (5-8)	0.132
Adenocarcinoma	11	3 (2-7)	
Adenosquamous carcinoma	3	3 (1-100)	
Other	3	3 (1-100)	
Primary treatment			
Radiation	43	5 (3-8)	0.517
Concurrent chemoradiation	47	6 (4-7)	
Surgery	8	7 (3-100)	
Symptoms			
No	5	2 (1-100)	0.003
Yes	93	6 (4-9)	
Time to bone recurrence			
<21 months	47	3 (2-6)	0.104
≥21 months	51	7 (5-9)	
Tumor spread			
Solitary	61	6 (5-7)	0.765
Multiple	37	5 (3-9)	
Sites of bone recurrence			
Pelvis	12	7 (5-9)	0.424
Nonpelvis	72	5 (3-7)	
Both	14	5 (2-9)	
Coexisting recurrence			
No	42	6 (4-11)	0.357
Yes	56	6 (4-7)	
Treatment of bone recurrence			
Radiation	54	6 (3-7)	
Chemotherapy	7	6 (2-8)	
Radiation-chemotherapy	14	10 (4-15)	
Supportive care	20	4 (1-10)	
Surgery radiation chemotherapy	3	4 (1-100)	0.090
Leukocyte count (*μ*L)			
<10000	29	7 (5-9)	0.001
≥10000	69	3(2-5)	
Hemoglobin (g/dL)			
<11	42	5 (4-7)	0.171
≥11	56	7 (4-11)	
Platelet count (*μ*L)			
<400000	32	7 (5-9)	0.002
≥400000	66	3 (2-6)	

CI, confidence interval; FIGO stage, International Federation of Gynecology and Obstetrics Staging System 2009.

**Table 3:** Multivariable clinical prognostic factors for overall survival.

	Hazard ratio	95% Confidence interval	*p*-Value
Age at diagnosis of bone recurrence (years)			
<60	2.48	(1.47-4.18)	0.001
≥60	1.00		
Time to bone recurrence			
<21 months	1.63	(1.04-2.55)	0.031
≥21 months	1.00		

## Data Availability

The data used to support the findings of this study are available upon request from the first author via thitiatjimakul@yahoo.com.
